# "Ierne's" Prodding Processes

**Published:** 1918-08-17

**Authors:** 


*To the Editor of* THE HOSPITAL.

### "IERNE'S" PRODDING PROCESSES.

*To the Editor of* THE HOSPITAL.

SIR,—Far be it from me to wish to cross swords with "Ierne"—as to whose sex even I am not sure—neither have I the pen of a ready writer, but if a member of our profession she (?) appears to view the average matron through the glasses of a probationer in the very early stage of her career, who is apt to think a matron's pastime confined chiefly to making irksome rules. I will refer to a few points touched upon recently, and which I have expected others more able than myself to question.

"Ierne" "cannot conceive of the average matron ever appearing before her board [is not committee more usual?] as the champion of liberality, even if she were convinced that justice was not being done." How unfortunate must her (?) experience of matrons have been, whether by hearsay, or in the flesh! In the numerous reports of increases in the salaries of various nursing staffs recently I feel quite safe in stating as a fact that it was the matron who voluntarily approached her committee with the suggestion.

Then, touching the "economist" point, I agree that unless a careful and economical manager the matron is unfit for her task, but between economy in food and general upkeep and the reasonable payment and comfort of her nurses there is a wide gulf, more especially as a fellow-feeling makes us wondrous kind, and she is frequently the last to be considered and seldom overpaid herself.

The *inarticulate* nurse is unfortunately almost general, but I think a more appropriate word might have been found than "agitator" for the *articulate* ones, and such an unkind fate as was the lot of the one specified cannot be taken as usual. I am not alone, I am sure, in wishing my nurses to think and act more independently than they do; but again there is another side even to this, which also bears on the objection made to so many matrons being members of the Council of the College of Nursing. Speaking from my own past experience, I doubt whether I should have felt either able *or* willing to venture an opinion on advanced nursing or administration before working by degrees through the different stages of a nurse's work and duties before being capable of filling a matron's post.

These personal opinions are probably only interesting to myself, but "Ierne" continues the prodding process week after week with no apparent effect, and in consequence is getting a little peevish, *I* think.—I am, yours faithfully, M. C. E.

